# Clinical Trials and Clinical Research: A Comprehensive Review

**DOI:** 10.7759/cureus.35077

**Published:** 2023-02-16

**Authors:** Venkataramana Kandi, Sabitha Vadakedath

**Affiliations:** 1 Clinical Microbiology, Prathima Institute of Medical Sciences, Karimnagar, IND; 2 Biochemistry, Prathima Institute of Medical Sciences, Karimnagar, IND

**Keywords:** ethical concerns, audit, clinical trials, therapeutic drug, efficacy, medical research, clinical research

## Abstract

Clinical research is an alternative terminology used to describe medical research. Clinical research involves people, and it is generally carried out to evaluate the efficacy of a therapeutic drug, a medical/surgical procedure, or a device as a part of treatment and patient management. Moreover, any research that evaluates the aspects of a disease like the symptoms, risk factors, and pathophysiology, among others may be termed clinical research. However, clinical trials are those studies that assess the potential of a therapeutic drug/device in the management, control, and prevention of disease. In view of the increasing incidences of both communicable and non-communicable diseases, and especially after the effects that Coronavirus Disease-19 (COVID-19) had on public health worldwide, the emphasis on clinical research assumes extremely essential. The knowledge of clinical research will facilitate the discovery of drugs, devices, and vaccines, thereby improving preparedness during public health emergencies. Therefore, in this review, we comprehensively describe the critical elements of clinical research that include clinical trial phases, types, and designs of clinical trials, operations of trial, audit, and management, and ethical concerns.

## Introduction and background

A clinical trial is a systematic process that is intended to find out the safety and efficacy of a drug/device in treating/preventing/diagnosing a disease or a medical condition [1,2]. Clinical trial includes various phases that include phase 0 (micro-dosing studies), phase 1, phase 2, phase 3, and phase 4 [3]. Phase 0 and phase 2 are called exploratory trial phases, phase 1 is termed the non-therapeutic phase, phase 3 is known as the therapeutic confirmatory phase, and phase 4 is called the post-approval or the post-marketing surveillance phase. Phase 0, also called the micro-dosing phase, was previously done in animals but now it is carried out in human volunteers to understand the dose tolerability (pharmacokinetics) before being administered as a part of the phase 1 trial among healthy individuals. The details of the clinical trial phases are shown in Table 1.

**Table 1:** The phases, types, and nature of clinical trial studies This table has been created by the authors. MTD: maximum tolerated dose; SAD: single ascending dose; MAD: multiple ascending doses; NDA: new drug application; FDA: food and drug administration

Clinical trial phase	Type of the study	Nature of study
Phase 0	Exploratory	Examines too low (1/100^th^) concentrations (micro-dosing) of the drug for less time. Study the pharmacokinetics and determine the dose for phase I studies. Previously done in animals but now it is carried out in humans.
Phase I, Phase Ia, Phase Ib	Non-therapeutic trial	Around <50 healthy subjects are recruited. Establishes a safe dose range, and the MTD. Examines the pharmacokinetic and pharmacodynamic effects. Usually single-center studies. Phase Ia: SAD, and MTD. Duration of one week to several months depending on the trial and includes 6-8 groups of 3-6 participants. Phase Ib: MAD and the dose is gradually narrowed down. Three groups of 8 individuals each.
Phase II, Phase IIa, Phase IIb	Exploratory trial	Recruiting around 5-100 patients of either sex. Examines the effective dosage and the therapeutic effects on patients. It decides the therapeutic regimen and drug-drug interactions. Usually, multicentre studies. Phase IIa: Decides the drug dosage, includes 20-30 patients, and takes up to weeks/months. Phase IIb: Studies dose-response relationship, drug-drug interactions, and comparison with a placebo.
Phase III	Therapeutic confirmatory trial	More than 300 patients (up to 3000) of either sex are recruited in this study and are multicentric trials. Pre-marketing phase examines the efficacy and the safety of the drug. Comparison of the test drug with the placebo/standard drug. Adverse drug reactions/adverse events are noted. Initiate the process of NDA with appropriate regulatory agencies like the FDA.
Phase IV	Post-approval study	After approval/post-licensure and post-marketing studies/surveillance studies. Following up on the patients for an exceptionally long time for potential adverse reactions and drug-drug interactions.

Clinical research design has two major types that include non-interventional/observational and interventional/experimental studies. The non-interventional studies may have a comparator group (analytical studies like case-control and cohort studies), or without it (descriptive study). The experimental studies may be either randomized or non-randomized. Clinical trial designs are of several types that include parallel design, crossover design, factorial design, randomized withdrawal approach, adaptive design, superiority design, and non-inferiority design. The advantages and disadvantages of clinical trial designs are depicted in Table 2.

**Table 2:** Clinical trial designs, their advantages, and disadvantages This table has been created by the authors.

Trial design type	Type of the study	Nature of study	Advantages/disadvantages
Parallel	Randomized	This is the most frequent design wherein each arm of the study group is allocated a particular treatment (placebo (an inert substance)/therapeutic drug)	The placebo arm does not receive the trial drug, so may not get the benefit of it
Crossover	Randomized	The patient in this trial gets each drug and the patients serve as a control themselves	Avoids participant bias in treatment and requires a small sample size. This design is not suitable for research on acute diseases.
Factorial	Non-randomized	Two or more interventions on the participants and the study can provide information on the interactions between the drugs	The study design is complex
Randomized withdrawal approach	Randomized	This study evaluates the time/duration of the drug therapy	The study uses a placebo to understand the efficacy of a drug in treating the disease
Matched pairs	Post-approval study	Recruit patients with the same characteristics	Less variability

**Figure 1:**
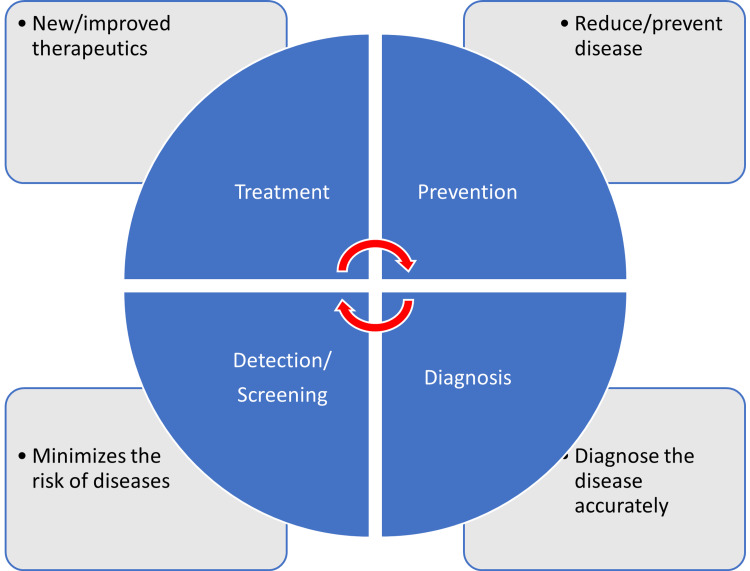
Pictorial representation of disease diagnosis, treatment, and prevention This figure has been created by the authors.

There are different types of clinical trials that include those which are conducted for treatment, prevention, early detection/screening, and diagnosis. These studies address the activities of an investigational drug on a disease and its outcomes [4]. They assess whether the drug is able to prevent the disease/condition, the ability of a device to detect/screen the disease, and the efficacy of a medical test to diagnose the disease/condition. The pictorial representation of a disease diagnosis, treatment, and prevention is depicted in Figure 1.

**Figure 2:**
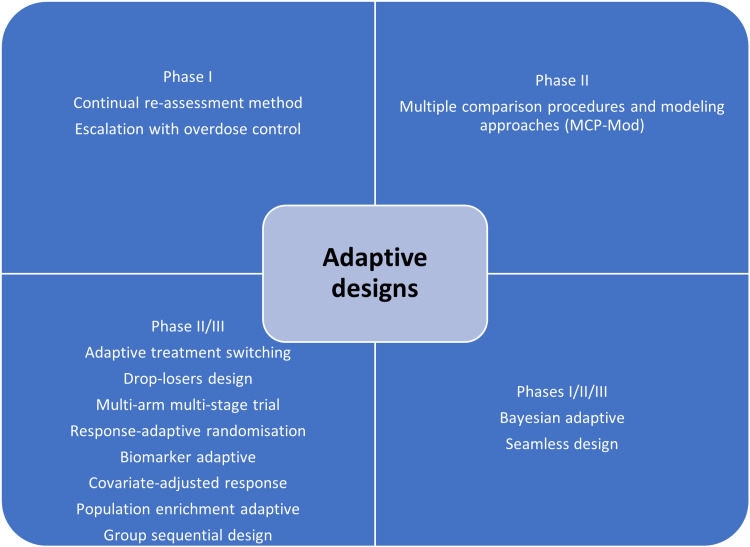
Pictorial representation of adaptive clinical trial designs This figure has been created by the authors.

The clinical trial designs could be improvised to make sure that the study's validity is maintained/retained. The adaptive designs facilitate researchers to improvise during the clinical trial without interfering with the integrity and validity of the results. Moreover, it allows flexibility during the conduction of trials and the collection of data. Despite these advantages, adaptive designs have not been universally accepted among clinical researchers. This could be attributed to the low familiarity of such designs in the research community. The adaptive designs have been applied during various phases of clinical trials and for different clinical conditions [5,6]. The adaptive designs applied during different phases are depicted in Figure 2.

The Bayesian adaptive trial design has gained popularity, especially during the Coronavirus Disease-19 (COVID-19) pandemic. Such designs could operate under a single master protocol. It operates as a platform trial wherein multiple treatments can be tested on different patient groups suffering from disease [7].

In this review, we comprehensively discuss the essential elements of clinical research that include the principles of clinical research, planning clinical trials, practical aspects of clinical trial operations, essentials of clinical trial applications, monitoring, and audit, clinical trial data analysis, regulatory audits, and project management, clinical trial operations at the investigation site, the essentials of clinical trial experiments involving epidemiological, and genetic studies, and ethical considerations in clinical research/trials.

## Review

A clinical trial involves the study of the effect of an investigational drug/any other intervention in a defined population/participant. The clinical research includes a treatment group and a placebo wherein each group is evaluated for the efficacy of the intervention (improved/not improved) [8].

Clinical trials are broadly classified into controlled and uncontrolled trials. The uncontrolled trials are potentially biased, and the results of such research are not considered as equally as the controlled studies. Randomized controlled trials (RCTs) are considered the most effective clinical trials wherein the bias is minimized, and the results are considered reliable. There are different types of randomizations and each one has clearly defined functions as elaborated in Table 3.

**Table 3:** Different types of randomizations in clinical trials This table has been created by the authors.

Randomization type	Functions
Simple randomization	The participants are assigned to a case or a control group based on flipping coin results/computer assignment
Block randomization	Equal and small groups of both cases and controls
Stratified randomization	Randomization based on the age of the participant and other covariates
Co-variate adaptive randomization/minimization	Sequential assignment of a new participant into a group based on the covariates
Randomization by body halves or paired organs (Split body trials)	One intervention is administered to one-half of the body and the comparator intervention is assigned to another half of the body
Clustered randomization	Intervention is administered to clusters/groups by randomization to prevent contamination and either active or comparator intervention is administered for each group
Allocation by randomized consent (Zelen trials)	Patients are allocated to one of the two trial arms

Principles of clinical trial/research

Clinical trials or clinical research are conducted to improve the understanding of the unknown, test a hypothesis, and perform public health-related research [2,3]. This is majorly carried out by collecting the data and analyzing it to derive conclusions. There are various types of clinical trials that are majorly grouped as analytical, observational, and experimental research. Clinical research can also be classified into non-directed data capture, directed data capture, and drug trials. Clinical research could be prospective or retrospective. It may also be a case-control study or a cohort study. Clinical trials may be initiated to find treatment, prevent, observe, and diagnose a disease or a medical condition.

Among the various types of clinical research, observational research using a cross-sectional study design is the most frequently performed clinical research. This type of research is undertaken to analyze the presence or absence of a disease/condition, potential risk factors, and prevalence and incidence rates in a defined population. Clinical trials may be therapeutic or non-therapeutic type depending on the type of intervention. The therapeutic type of clinical trial uses a drug that may be beneficial to the patient. Whereas in a non-therapeutic clinical trial, the participant does not benefit from the drug. The non-therapeutic trials provide additional knowledge of the drug for future improvements. Different terminologies of clinical trials are delineated in Table 4.

**Table 4:** Clinical trial methods and terminologies This table has been created by the authors.

Type of clinical trial	Definition
Randomized trial	Study participants are randomly assigned to a group
Open-label	Both study subjects and the researchers are aware of the drug being tested
Blinded (single-blind)	In single-blind studies, the subject has no idea about the group (test/control) in which they are placed
Double-blind (double-blind)	In the double-blind study, the subjects as well as the investigator have no idea about the test/control group
Placebo	A substance that appears like a drug but has no active moiety
Add-on	An additional drug apart from the clinical trial drug given to a group of study participants
Single center	A study being carried out at a particular place/location/center
Multi-center	A study is being carried out at multiple places/locations/centers

In view of the increased cost of the drug discovery process, developing, and low-income countries depend on the production of generic drugs. The generic drugs are similar in composition to the patented/branded drug. Once the patent period is expired generic drugs can be manufactured which have a similar quality, strength, and safety as the patented drug [9]. The regulatory requirements and the drug production process are almost the same for the branded and the generic drug according to the Food and Drug Administration (FDA), United States of America (USA).

The bioequivalence (BE) studies review the absorption, distribution, metabolism, and excretion (ADME) of the generic drug. These studies compare the concentration of the drug at the desired location in the human body, called the peak concentration of the drug (Cmax). The extent of absorption of the drug is measured using the area under the receiver operating characteristic curve (AUC), wherein the generic drug is supposed to demonstrate similar ADME activities as the branded drug. The BE studies may be undertaken in vitro (fasting, non-fasting, sprinkled fasting) or in vivo studies (clinical, bioanalytical, and statistical) [9].

Planning clinical trial/research

The clinical trial process involves protocol development, designing a case record/report form (CRF), and functioning of institutional review boards (IRBs). It also includes data management and the monitoring of clinical trial site activities. The CRF is the most significant document in a clinical study. It contains the information collected by the investigator about each subject participating in a clinical study/trial. According to the International Council for Harmonisation (ICH), the CRF can be printed, optical, or an electronic document that is used to record the safety and efficacy of the pharmaceutical drug/product in the test subjects. This information is intended for the sponsor who initiates the clinical study [10].

The CRF is designed as per the protocol and later it is thoroughly reviewed for its correctness (appropriate and structured questions) and finalized. The CRF then proceeds toward the print taking the language of the participating subjects into consideration. Once the CRF is printed, it is distributed to the investigation sites where it is filled with the details of the participating subjects by the investigator/nurse/subject/guardian of the subject/technician/consultant/monitors/pharmacist/pharmacokinetics/contract house staff. The filled CRFs are checked for their completeness and transported to the sponsor [11].

Effective planning and implementation of a clinical study/trial will influence its success. The clinical study majorly includes the collection and distribution of the trial data, which is done by the clinical data management section. The project manager is crucial to effectively plan, organize, and use the best processes to control and monitor the clinical study [10,11].

The clinical study is conducted by a sponsor or a clinical research organization (CRO). A perfect protocol, time limits, and regulatory requirements assume significance while planning a clinical trial. What, when, how, and who are clearly planned before the initiation of a study trial. Regular review of the project using the bar and Gantt charts, and maintaining the timelines assume increased significance for success with the product (study report, statistical report, database) [10,11].

The steps critical to planning a clinical trial include the idea, review of the available literature, identifying a problem, formulating the hypothesis, writing a synopsis, identifying the investigators, writing a protocol, finding a source of funding, designing a patient consent form, forming ethics boards, identifying an organization, preparing manuals for procedures, quality assurance, investigator training and initiation of the trial by recruiting the participants [10].

The two most important points to consider before the initiation of the clinical trial include whether there is a need for a clinical trial, if there is a need, then one must make sure that the study design and methodology are strong for the results to be reliable to the people [11].

For clinical research to envisage high-quality results, the study design, implementation of the study, quality assurance in data collection, and alleviation of bias and confounding factors must be robust [12]. Another important aspect of conducting a clinical trial is improved management of various elements of clinical research that include human and financial resources. The role of a trial manager to make a successful clinical trial was previously reported. The trial manager could play a key role in planning, coordinating, and successfully executing the trial. Some qualities of a trial manager include better communication and motivation, leadership, and strategic, tactical, and operational skills [13].

Practical aspects of a clinical trial operations

There are different types of clinical research. Research in the development of a novel drug could be initiated by nationally funded research, industry-sponsored research, and clinical research initiated by individuals/investigators. According to the documents 21 code of federal regulations (CFR) 312.3 and ICH E-6 Good Clinical Practice (GCP) 1.54, an investigator is an individual who initiates and conducts clinical research [14]. The investigator plan, design, conduct, monitor, manage data, compile reports, and supervise research-related regulatory and ethical issues. To manage a successful clinical trial project, it is essential for an investigator to give the letter of intent, write a proposal, set a timeline, develop a protocol and related documents like the case record forms, define the budget, and identify the funding sources.

Other major steps of clinical research include the approval of IRBs, conduction and supervision of the research, data review, and analysis. Successful clinical research includes various essential elements like a letter of intent which is the evidence that supports the interest of the researcher to conduct drug research, timeline, funding source, supplier, and participant characters.

Quality assurance, according to the ICH and GCP guidelines, is necessary to be implemented during clinical research to generate quality and accurate data. Each element of the clinical research must have been carried out according to the standard operating procedure (SOP), which is written/determined before the initiation of the study and during the preparation of the protocol [15].

The audit team (quality assurance group) is instrumental in determining the authenticity of the clinical research. The audit, according to the ICH and GCP, is an independent and external team that examines the process (recording the CRF, analysis of data, and interpretation of data) of clinical research. The quality assurance personnel are adequately trained, become trainers if needed, should be good communicators, and must handle any kind of situation. The audits can be at the investigator sites evaluating the CRF data, the protocol, and the personnel involved in clinical research (source data verification, monitors) [16].

Clinical trial operations are governed by legal and regulatory requirements, based on GCPs, and the application of science, technology, and interpersonal skills [17]. Clinical trial operations are complex, time and resource-specific that requires extensive planning and coordination, especially for the research which is conducted at multiple trial centers [18].

Recruiting the clinical trial participants/subjects is the most significant aspect of clinical trial operations. Previous research had noted that most clinical trials do not meet the participant numbers as decided in the protocol. Therefore, it is important to identify the potential barriers to patient recruitment [19].

Most clinical trials demand huge costs, increased timelines, and resources. Randomized clinical trial studies from Switzerland were analyzed for their costs which revealed approximately 72000 USD for a clinical trial to be completed. This study emphasized the need for increased transparency with respect to the costs associated with the clinical trial and improved collaboration between collaborators and stakeholders [20].

Clinical trial applications, monitoring, and audit

Among the most significant aspects of a clinical trial is the audit. An audit is a systematic process of evaluating the clinical trial operations at the site. The audit ensures that the clinical trial process is conducted according to the protocol, and predefined quality system procedures, following GCP guidelines, and according to the requirements of regulatory authorities [21].

The auditors are supposed to be independent and work without the involvement of the sponsors, CROs, or personnel at the trial site. The auditors ensure that the trial is conducted by designated professionally qualified, adequately trained personnel, with predefined responsibilities. The auditors also ensure the validity of the investigational drug, and the composition, and functioning of institutional review/ethics committees. The availability and correctness of the documents like the investigational broacher, informed consent forms, CRFs, approval letters of the regulatory authorities, and accreditation of the trial labs/sites [21].

The data management systems, the data collection software, data backup, recovery, and contingency plans, alternative data recording methods, security of the data, personnel training in data entry, and the statistical methods used to analyze the results of the trial are other important responsibilities of the auditor [21,22].

According to the ICH-GCP Sec 1.29 guidelines the inspection may be described as an act by the regulatory authorities to conduct an official review of the clinical trial-related documents, personnel (sponsor, investigator), and the trial site [21,22]. The summary report of the observations of the inspectors is performed using various forms as listed in Table 5.

**Table 5:** The FDA regulatory forms for the submission of inspection results This table has been created by the authors. FDA: Food and Drug Administration; IND: investigational new drug; NDA: new drug application; IRB: institutional review board; CFR: code of federal regulations

Regulatory (FDA) form number	Components of the form
483	List of objectionable conditions/processes prepared by the FDA investigator and submitted to the auditee at the end of the inspection
482	The auditors submit their identity proofs and notice of inspections to the clinical investigators and later document their observations
1571	This document details the fact that the clinical trial is not initiated before 30 days of submitting the IND to the FDA for approval. The form confirms that the IRB complies with 21 CFR Part 56. The form details the agreement to follow regulatory requirements and names all the individuals who monitor the conduct and progress of the study and evaluate the safety of the clinical trial
1572	This form details the fact that the study is conducted after ethics approval ensures that the study is carried out according to protocol, informed consent, and IRB approval

Because protecting data integrity, the rights, safety, and well-being of the study participants are more significant while conducting a clinical trial, regular monitoring and audit of the process appear crucial. Also, the quality of the clinical trial greatly depends on the approach of the trial personnel which includes the sponsors and investigators [21].

The responsibility of monitoring lies in different hands, and it depends on the clinical trial site. When the trial is initiated by a pharmaceutical industry, the responsibility of trial monitoring depends on the company or the sponsor, and when the trial is conducted by an academic organization, the responsibility lies with the principal investigator [21].

An audit is a process conducted by an independent body to ensure the quality of the study. Basically, an audit is a quality assurance process that determines if a study is carried out by following the SPOs, in compliance with the GCPs recommended by regulatory bodies like the ICH, FDA, and other local bodies [21].

An audit is performed to review all the available documents related to the IRB approval, investigational drug, and the documents related to the patient care/case record forms. Other documents that are audited include the protocol (date, sign, treatment, compliance), informed consent form, treatment response/outcome, toxic response/adverse event recording, and the accuracy of data entry [22].

Clinical trial data analysis, regulatory audits, and project management

The essential elements of clinical trial management systems (CDMS) include the management of the study, the site, staff, subject, contracts, data, and document management, patient diary integration, medical coding, monitoring, adverse event reporting, supplier management, lab data, external interfaces, and randomization. The CDMS involves setting a defined start and finishing time, defining study objectives, setting enrolment and termination criteria, commenting, and managing the study design [23].

Among the various key application areas of clinical trial systems, the data analysis assumes increased significance. The clinical trial data collected at the site in the form of case record form is stored in the CDMS ensuring the errors with respect to the double data entry are minimized.

Clinical trial data management uses medical coding, which uses terminologies with respect to the medications and adverse events/serious adverse events that need to be entered into the CDMS. The project undertaken to conduct the clinical trial must be predetermined with timelines and milestones. Timelines are usually set for the preparation of protocol, designing the CRF, planning the project, identifying the first subject, and timelines for recording the patient’s data for the first visit.

The timelines also are set for the last subject to be recruited in the study, the CRF of the last subject, and the locked period after the last subject entry. The planning of the project also includes the modes of collection of the data, the methods of the transport of the CRFs, patient diaries, and records of severe adverse events, to the central data management sites (fax, scan, courier, etc.) [24].

The preparation of SOPs and the type and timing of the quality control (QC) procedures are also included in the project planning before the start of a clinical study. Review (budget, resources, quality of process, assessment), measure (turnaround times, training issues), and control (CRF collection and delivery, incentives, revising the process) are the three important aspects of the implementation of a clinical research project.

In view of the increasing complexity related to the conduct of clinical trials, it is important to perform a clinical quality assurance (CQA) audit. The CQA audit process consists of a detailed plan for conducting audits, points of improvement, generating meaningful audit results, verifying SOP, and regulatory compliance, and promoting improvement in clinical trial research [25]. All the components of a CQA audit are delineated in Table 6.

**Table 6:** Types of clinical trial audits This table has been created by the authors. CRF: case report form; CSR: clinical study report; IC: informed consent; PV: pharmacovigilance; SAE: serious adverse event

Product-specific audits program	Pharmacovigilance audits program
Protocol, CRF, IC, CSR	
Supplier	Safety data management
Clinical database	
Investigator site	Communications and regulatory reporting
Clinical site visit	
Study management	Signal detection and evaluation
SAE reporting	
Supplier audits program	Risk management and PV planning
Supplier qualification	
Sponsor data audit during the trial	Computerized system
Preferred vendor list after the trials	
Process/System audits program	Suppliers
Clinical safety reporting	
Data management	Regulatory inspection management program
Clinical supply	
Study monitoring	Assist with the audit response
Computerized system	Pre-inspection audit

Clinical trial operations at the investigator's site

The selection of an investigation site is important before starting a clinical trial. It is essential that the individuals recruited for the study meet the inclusion criteria of the trial, and the investigator's and patient's willingness to accept the protocol design and the timelines set by the regulatory authorities including the IRBs.

Before conducting clinical research, it is important for an investigator to agree to the terms and conditions of the agreement and maintain the confidentiality of the protocol. Evaluation of the protocol for the feasibility of its practices with respect to the resources, infrastructure, qualified and trained personnel available, availability of the study subjects, and benefit to the institution and the investigator is done by the sponsor during the site selection visit.

The standards of a clinical research trial are ensured by the Council for International Organizations of Medical Sciences (CIOMS), National Bioethics Advisory Commission (NBAC), United Nations Programme on Human Immunodeficiency Virus/Acquired Immunodeficiency Syndrome (HIV/AIDS) (UNAIDS), and World Medical Association (WMA) [26].

Recommendations for conducting clinical research based on the WMA support the slogan that says, “The health of my patient will be my first consideration.” According to the International Code of Medical Ethics (ICME), no human should be physically or mentally harmed during the clinical trial, and the study should be conducted in the best interest of the person [26].

Basic principles recommended by the Helsinki declaration include the conduction of clinical research only after the prior proof of the safety of the drug in animal and lab experiments. The clinical trials must be performed by scientifically, and medically qualified and well-trained personnel. Also, it is important to analyze the benefit of research over harm to the participants before initiating the drug trials.

The doctors may prescribe a drug to alleviate the suffering of the patient, save the patient from death, and gain additional knowledge of the drug only after obtaining informed consent. Under the equipoise principle, the investigators must be able to justify the treatment provided as a part of the clinical trial, wherein the patient in the placebo arm may be harmed due to the unavailability of the therapeutic/trial drug.

Clinical trial operations greatly depend on the environmental conditions and geographical attributes of the trial site. It may influence the costs and targets defined by the project before the initiation. It was noted that one-fourth of the clinical trial project proposals/applications submit critical data on the investigational drug from outside the country. Also, it was noted that almost 35% of delays in clinical trials owing to patient recruitment with one-third of studies enrolling only 5% of the participants [27].

It was suggested that clinical trial feasibility assessment in a defined geographical region may be undertaken for improved chances of success. Points to be considered under the feasibility assessment program include if the disease under the study is related to the population of the geographical region, appropriateness of the study design, patient, and comparator group, visit intervals, potential regulatory and ethical challenges, and commitments of the study partners, CROs in respective countries (multi-centric studies) [27].

Feasibility assessments may be undertaken at the program level (ethics, regulatory, and medical preparedness), study level (clinical, regulatory, technical, and operational aspects), and at the investigation site (investigational drug, competency of personnel, participant recruitment, and retention, quality systems, and infrastructural aspects) [27].

Clinical trials: true experiments

In accordance with the revised schedule "Y" of the Drugs and Cosmetics Act (DCA) (2005), a drug trial may be defined as a systematic study of a novel drug component. The clinical trials aim to evaluate the pharmacodynamic, and pharmacokinetic properties including ADME, efficacy, and safety of new drugs.

According to the drug and cosmetic rules (DCR), 1945, a new chemical entity (NCE) may be defined as a novel drug approved for a disease/condition, in a specified route, and at a particular dosage. It also may be a new drug combination, of previously approved drugs.

A clinical trial may be performed in three types; one that is done to find the efficacy of an NCE, a comparison study of two drugs against a medical condition, and the clinical research of approved drugs on a disease/condition. Also, studies of the bioavailability and BE studies of the generic drugs, and the drugs already approved in other countries are done to establish the efficacy of new drugs [28].

Apart from the discovery of a novel drug, clinical trials are also conducted to approve novel medical devices for public use. A medical device is defined as any instrument, apparatus, appliance, software, and any other material used for diagnostic/therapeutic purposes. The medical devices may be divided into three classes wherein class I uses general controls; class II uses general and special controls, and class III uses general, special controls, and premarket approvals [28].

The premarket approval applications ensure the safety and effectiveness, and confirmation of the activities from bench to animal to human clinical studies. The FDA approval for investigational device exemption (IDE) for a device not approved for a new indication/disease/condition. There are two types of IDE studies that include the feasibility study (basic safety and potential effectiveness) and the pivotal study (trial endpoints, randomization, monitoring, and statistical analysis plan) [28].

As evidenced by the available literature, there are two types of research that include observational and experimental research. Experimental research is alternatively known as the true type of research wherein the research is conducted by the intervention of a new drug/device/method (educational research). Most true experiments use randomized control trials that remove bias and neutralize the confounding variables that may interfere with the results of research [28].

The variables that may interfere with the study results are independent variables also called prediction variables (the intervention), dependent variables (the outcome), and extraneous variables (other confounding factors that could influence the outside). True experiments have three basic elements that include manipulation (that influence independent variables), control (over extraneous influencers), and randomization (unbiased grouping) [29].

Experiments can also be grouped as true, quasi-experimental, and non-experimental studies depending on the presence of specific characteristic features. True experiments have all three elements of study design (manipulation, control, randomization), and prospective, and have great scientific validity. Quasi-experiments generally have two elements of design (manipulation and control), are prospective, and have moderate scientific validity. The non-experimental studies lack manipulation, control, and randomization, are generally retrospective, and have low scientific validity [29].

Clinical trials: epidemiological and human genetics study

Epidemiological studies are intended to control health issues by understanding the distribution, determinants, incidence, prevalence, and impact on health among a defined population. Such studies are attempted to perceive the status of infectious diseases as well as non-communicable diseases [30].

Experimental studies are of two types that include observational (cross-sectional studies (surveys), case-control studies, and cohort studies) and experimental studies (randomized control studies) [3,31]. Such research may pose challenges related to ethics in relation to the social and cultural milieu.

Biomedical research related to human genetics and transplantation research poses an increased threat to ethical concerns, especially after the success of the human genome project (HGP) in the year 2000. The benefits of human genetic studies are innumerable that include the identification of genetic diseases, in vitro fertilization, and regeneration therapy. Research related to human genetics poses ethical, legal, and social issues (ELSI) that need to be appropriately addressed. Most importantly, these genetic research studies use advanced technologies which should be equally available to both economically well-placed and financially deprived people [32].

Gene therapy and genetic manipulations may potentially precipitate conflict of interest among the family members. The research on genetics may be of various types that include pedigree studies (identifying abnormal gene carriers), genetic screening (for diseases that may be heritable by the children), gene therapeutics (gene replacement therapy, gene construct administration), HGP (sequencing the whole human genome/deoxyribonucleic acid (DNA) fingerprinting), and DNA, cell-line banking/repository [33]. The biobanks are established to collect and store human tissue samples like umbilical tissue, cord blood, and others [34].

Epidemiological studies on genetics are attempts to understand the prevalence of diseases that may be transmitted among families. The classical epidemiological studies may include single case observations (one individual), case series (< 10 individuals), ecological studies (population/large group of people), cross-sectional studies (defined number of individuals), case-control studies (defined number of individuals), cohort (defined number of individuals), and interventional studies (defined number of individuals) [35].

Genetic studies are of different types that include familial aggregation (case-parent, case-parent-grandparent), heritability (study of twins), segregation (pedigree study), linkage study (case-control), association, linkage, disequilibrium, cohort case-only studies (related case-control, unrelated case-control, exposure, non-exposure group, case group), cross-sectional studies, association cohort (related case-control, familial cohort), and experimental retrospective cohort (clinical trial, exposure, and non-exposure group) [35].

Ethics and concerns in clinical trial/research

Because clinical research involves animals and human participants, adhering to ethics and ethical practices assumes increased significance [36]. In view of the unethical research conducted on war soldiers after the Second World War, the Nuremberg code was introduced in 1947, which promulgated rules for permissible medical experiments on humans. The Nuremberg code suggests that informed consent is mandatory for all the participants in a clinical trial, and the study subjects must be made aware of the nature, duration, and purpose of the study, and potential health hazards (foreseen and unforeseen). The study subjects should have the liberty to withdraw at any time during the trial and to choose a physician upon medical emergency. The other essential principles of clinical research involving human subjects as suggested by the Nuremberg code included benefit to the society, justification of study as noted by the results of the drug experiments on animals, avoiding even minimal suffering to the study participants, and making sure that the participants don’t have life risk, humanity first, improved medical facilities for participants, and suitably qualified investigators [37].

During the 18th world medical assembly meeting in the year 1964, in Helsinki, Finland, ethical principles for doctors practicing research were proposed. Declaration of Helsinki, as it is known made sure that the interests and concerns of the human participants will always prevail over the interests of the society. Later in 1974, the National Research Act was proposed which made sure that the research proposals are thoroughly screened by the Institutional ethics/Review Board. In 1979, the April 18th Belmont report was proposed by the national commission for the protection of human rights during biomedical and behavioral research. The Belmont report proposed three core principles during research involving human participants that include respect for persons, beneficence, and justice. The ICH laid down GCP guidelines [38]. These guidelines are universally followed throughout the world during the conduction of clinical research involving human participants.

ICH was first founded in 1991, in Brussels, under the umbrella of the USA, Japan, and European countries. The ICH conference is conducted once every two years with the participation from the member countries, observers from the regulatory agencies, like the World Health Organization (WHO), European Free Trade Association (EFTA), and the Canadian Health Protection Branch, and other interested stakeholders from the academia and the industry. The expert working groups of the ICH ensure the quality, efficacy, and safety of the medicinal product (drug/device). Despite the availability of the Nuremberg code, the Belmont Report, and the ICH-GCP guidelines, in the year 1982, International Ethical Guidelines for Biomedical Research Involving Human Subjects was proposed by the CIOMS in association with WHO [39]. The CIOMS protects the rights of the vulnerable population, and ensures ethical practices during clinical research, especially in underdeveloped countries [40]. In India, the ethical principles for biomedical research involving human subjects were introduced by the Indian Council of Medical Research (ICMR) in the year 2000 and were later amended in the year 2006 [41]. Clinical trial approvals can only be done by the IRB approved by the Drug Controller General of India (DGCI) as proposed in the year 2013 [42].

Current perspectives and future implications

A recent study attempted to evaluate the efficacy of adaptive clinical trials in predicting the success of a clinical trial drug that entered phase 3 and minimizing the time and cost of drug development. This study highlighted the drawbacks of such clinical trial designs that include the possibility of type 1 (false positive) and type 2 (false negative) errors [43].

The usefulness of animal studies during the preclinical phases of a clinical trial was evaluated in a previous study which concluded that animal studies may not completely guarantee the safety of the investigational drug. This is noted by the fact that many drugs which passed toxicity tests in animals produced adverse reactions in humans [44].

The significance of BE studies to compare branded and generic drugs was reported previously. The pharmacokinetic BE studies of Amoxycillin comparing branded and generic drugs were carried out among a group of healthy participants. The study results have demonstrated that the generic drug had lower Cmax as compared to the branded drug [45].

To establish the BE of the generic drugs, randomized crossover trials are carried out to assess the Cmax and the AUC. The ratio of each pharmacokinetic characteristic must match the ratio of AUC and/or Cmax, 1:1=1 for a generic drug to be considered as a bioequivalent to a branded drug [46].

Although the generic drug development is comparatively more beneficial than the branded drugs, synthesis of extended-release formulations of the generic drug appears to be complex. Since the extended-release formulations remain for longer periods in the stomach, they may be influenced by gastric acidity and interact with the food. A recent study suggested the use of bio-relevant dissolution tests to increase the successful production of generic extended-release drug formulations [47].

Although RCTs are considered the best designs, which rule out bias and the data/results obtained from such clinical research are the most reliable, RCTs may be plagued by miscalculation of the treatment outcomes/bias, problems of cointerventions, and contaminations [48].

The perception of healthcare providers regarding branded drugs and their view about the generic equivalents was recently analyzed and reported. It was noted that such a perception may be attributed to the flexible regulatory requirements for the approval of a generic drug as compared to a branded drug. Also, could be because a switch from a branded drug to a generic drug in patients may precipitate adverse events as evidenced by previous reports [49].

Because the vulnerable population like drug/alcohol addicts, mentally challenged people, children, geriatric age people, military persons, ethnic minorities, people suffering from incurable diseases, students, employees, and pregnant women cannot make decisions with respect to participating in a clinical trial, ethical concerns, and legal issues may prop up, that may be appropriately addressed before drug trials which include such groups [50].

## Conclusions

Clinical research and clinical trials are important from the public health perspective. Clinical research facilitates scientists, public health administrations, and people to increase their understanding and improve preparedness with reference to the diseases prevalent in different geographical regions of the world. Moreover, clinical research helps in mitigating health-related problems as evidenced by the current Severe Acute Respiratory Syndrome Coronavirus-2 (SARS-CoV-2) pandemic and other emerging and re-emerging microbial infections. Clinical trials are crucial to the development of drugs, devices, and vaccines. Therefore, scientists are required to be up to date with the process and procedures of clinical research and trials as discussed comprehensively in this review.
